# Functional conservation and divergence in plant-specific *GRF* gene family revealed by sequences and expression analysis

**DOI:** 10.1515/biol-2022-0018

**Published:** 2022-03-11

**Authors:** Lingyan Meng, Xiaomeng Li, Yue Hou, Yaxuan Li, Yingkao Hu

**Affiliations:** College of Life Sciences, Capital Normal University, Beijing 100048, China

**Keywords:** growth regulatory factors, phylogenetic analysis, positive selection, functional divergence, expression profile

## Abstract

Unique to plants, growth regulatory factors (GRFs) play important roles in plant growth and reproduction. This study investigated the evolutionary and functional characteristics associated with plant growth. Using genome-wide analysis of 15 plant species, 173 members of the *GRF* family were identified and phylogenetically categorized into six groups. All members contained WRC and QLQ conserved domains, and the family’s expansion largely depended on segmental duplication. The promoter region of the *GRF* gene family mainly contained four types of *cis*-acting elements (light-responsive elements, development-related elements, hormone-responsive elements, and environmental stress-related elements) that are mainly related to gene expression levels. Functional divergence analysis revealed that changes in amino acid site evolution rate played a major role in the differentiation of the *GRF* gene family, with ten significant sites identified. Six significant sites were identified for positive selection. Moreover, the four groups of coevolutionary sites identified may play a key role in regulating the transcriptional activation of the GRF protein. Expression profiles revealed that *GRF* genes were generally highly expressed in young plant tissues and had tissue or organ expression specificity, demonstrating their functional conservation with distinct divergence. The results of these sequence and expression analyses are expected to provide molecular evolutionary and functional references for the plant *GRF* gene family.

## 1 Introduction

Transcription factors (TFs), also known as *trans*-acting factors, bind to DNA in a sequence-specific manner and regulate transcription. They are the main regulators of gene expression and play an important role in plant growth and development, response and adaptation to various stresses, and defense responses [1,2]. Growth regulatory factors (GRFs) are plant-specific TFs that were first discovered in rice (*Oryza sativa* L.) intercalary meristems named *OsGRF1* that play a regulatory role in gibberellic acid (GA)-induced stem extension [3,4]. Subsequently, *GRF* members were continuously discovered in other plant species, such as *Arabidopsis thaliana* [5], corn [6], cabbage [7], tomato [8], and wheat [9].

GRF proteins contain conserved WRC (Trp, Arg, and Cys) and QLQ (Gln, Leu, and Gln) domains at the N-terminal [4,7,10,11]. The WRC domain contains a nuclear localization signal and a C_3_H zinc finger structure, similar to the plant-specific Cys–Cys–Cys–His (CX_9_CX_10_CX_2_H) motif, and participates in DNA binding [3,12,13]. The QLQ domain is composed of a highly conserved Gln–Leu–Gln (QX_3_LX_2_Q) residue and neighboring residues that are similar to the N-terminal of the chromatin remodeling complex (SWI2/SNF2) in yeast and can interact with SNF11 [14]. In addition, the lengths and amino acid sequences of the C-terminal of GRF proteins are different, but they still have the common characteristics of TFs [11]. Studies have shown that the lack of complete C-terminal will lead to the loss of transcriptional activation activity, which will affect plant growth [6], and there are TQL, GGPL, and FFD domains at the C-terminus of partial plants. The QLQ domain also participates in the interaction with the SYT N-terminal homology (SNH) domain at the N-terminal of the GRF-interacting factor (GIF) protein to form a transcriptional activator [15]. As a transcriptional coactivator, GIF promotes gene alteration of the GRF protein. *GRF* and *GIF* genes are highly expressed in almost all meristems [16,17] and participate in regulating the growth and development of some tissues and organs in plants; interestingly, GRF/GIF-mediated growth is usually associated with enhanced cell proliferation or cell expansion [15,18]. Previous studies have shown that microRNA396 (miR396) is involved in the regulation of *GRF* gene expression. MiR396 regulates most GRF members after transcription and fine-tunes their expression to control the GRF/GIF-dependent process [19,20]. Kim [16] showed that miR396 could regulate the proliferation of cells and the size of meristems.

In the model plant *Arabidopsis*, *AtGRF1*, *2*, and *3* control the size of plant leaves by reorganizing the size of plant cells. For instance, compared with the wild type, overexpression of *AtGRF1* and *AtGRF2* leads to larger leaves and cotyledons or leads to a late flowering phenotype [11,21]. *GRF4* is not only involved in the expansion and growth of leaf cells but is also necessary for the development of cotyledons and shoot apex meristems [22]. Importantly, a study demonstrated that *GRF5* could promote the duration of the cell proliferation phase during leaf development [23]. Similarly, GIF1 coordinates cell proliferation in different cell layers in rice by translocating through plasmodesmata [16]. Moreover, *GRF* participates in the osmotic resistance of plants, and *AtGRF7* mutants are more resistant to drought and salt stress than wild type and *AtGRF7* overexpression lines [13]. Additionally, the regulation mechanism of GRF expression is complex. The miR396/GRF regulatory network has an adjusted effect on flower organ development [24]. In tomatoes, miR396 has two mature types (miR396a and miR396b). The study by Cao et al. found that downregulation of miR396a and miR396b results in an overall upregulation of target GRFs, resulting in the obvious enlargement of flowers, sepals, and fruits [8]. Furthermore, miR396–GRF/GIF has been important in regulating plant senescence and affects different stages of leaf development. It can coordinate plant growth and physiological responses with endogenous and environmental signals [18]. *AtGRF3* lines with mutations in miR396 binding site and lines overexpressing *AtGRF5* have delayed leaf senescence [25]. In *Arabidopsis*, ectopic overexpression of miR396 inhibits the expression of *GRF* genes and inhibits the transcriptional coactivator GIF1. The transcriptional expression of GRFs was also regulated by GA3, which enhances the expression of *OsGRF1*, *2*, *3*, *7*, *8*, *10*, and *12* [10]. Studies have shown that GRF protein can also affect plant growth and development by negatively regulating the expression of dehydration-response element-binding (DREB2A) protein and knotted-like homeobox protein [26,27]. GRFs also participate in plant ear development [28], root growth [29,30], floral organogenesis [31], apical meristem growth and maintenance [26,32], and other processes.

To better understand the dynamics of the evolution of the *GRF* gene and the functional relationship between gene family members, we constructed a phylogenetic tree of 15 plants species. Additionally, we conducted an analysis of gene duplication methods once tandem and segment duplication events may lead to the generation of new gene family members. Of note, we also looked for positive selection sites and coevolution sites, usually related to protein functions in the protein evolution process. The expression profile obtained through the transcriptome data reflects the conservation and difference of *GRF* genes in different tissues. The organizational differences in the *cis*-acting regulatory elements in the promoter region can partially explain the differences in their expression.

## 2 Materials and methods

### 2.1 Identification of *GRF* gene family

The *A. thaliana GRF* gene sequences were downloaded from the TAIR database (http://www.arabidopsis.org/) and used as seed sequences to identify *GRF* gene family members in 15 sequenced plant species (*Brachypodium distachyon*, *O. sativa*, *Sorghum bicolor*, *Zea mays*, *Physcomitrella patens*, *Selaginella moellendorffii*, *A. thaliana*, *Brassica rapa*, *Citrus sinensis*, *Glycine max*, *Gossypium raimondii*, *Medicago sativa*, *Phaseolus vulgaris*, *Populus trichocarpa*, *and Solanum lycopersicum*) using the BLASTP tool in the Phytozome database (http://www.phytozome.org). These 15 species represent the plant kingdom from lower to higher plants. An *E*-value ≤ 1 × 10^−5^ and a complete open reading frame were used as the selection criteria for protein sequences. Pfam (http://pfam.xfam.org) and SMART (http://smart.embl-heidelberg.de/) proteomics service programs were used to verify whether the candidate GRF protein contains conserved WRC(PF08879) and QLQ(PF08880) domains. In addition, the protein sequences, coding sequences (CDS), genomic sequences, and 2,000 bp sequences upstream of the start codon were obtained from the Phytozome database. The amino acid number, isoelectric point (pI), and molecular weight (MW) of GRF proteins were obtained from the ExPASy database (https://www.expasy.org/) [33].

### 2.2 Phylogenetic tree construction and analysis of exon–intron structure, motif, and *cis*-acting elements

Multiple sequence alignment of all amino acid sequences of the identified full-length GRF proteins was conducted using the MUSCLE program [34,35]. Based on the multiple sequence alignment files, MEGA7.0 was used to build phylogenetic trees under the default parameters using neighbor-joining (NJ) and maximum likelihood (ML) methods, with bootstrap set to 1,000 [36]. In addition, MrBayes 3.2.5 software was used to build a Bayesian evolutionary tree [37].

The exon–intron structure of GRFs was derived from the online tool GSDS (http://gsds.cbi.pku.edu.cn/) by comparing the CDS and genomic sequences [38]. Conserved motifs were detected using the MEME program (http://meme-suite.org/tools/meme) [39], which was run with the maximum number of motifs set to 20, whereas the remaining parameters were preset by the system.

The 2,000-bp upstream sequence of the start codon was collected from the Phytozome database (www.phytozome.net), and *cis*-acting elements of known sequences were analyzed using the PlantCARE online service platform (http://bioinformatics.psb.ugent.be/webtools/plantcare/html/) [40]. Five members (*orange1.1g047108m*, *orange1.1g028751m*, *orange1.1g007514m*, *Brara.I03590*, *Solyc10g083510*) with incomplete promoter sequences were manually deleted to exclude them from subsequent analyses.

### 2.3 Duplication event analysis

The synonymous substitution rates (*K*_s_) of *GRF* gene pairs produced by duplication events were identified using the Plant Genome Duplication Database (http://chibba.agtec.uga.edu/duplication), and segmental duplicated gene pairs with *K*_s_ values greater than one and anchor loci less than three were excluded [41,42]. The *K*-estimator method was used to calculate the *K*_s_, *K*_a_, and *K*_a_/*K*_s_ [43]. The approximate date of segmental duplication events was estimated using *T* = *K*_s_/2*λ* formula. According to earlier researches, the *λ* value of the approximate date used for the calculation of the duplication events are as follows: 1.5 × 10^−8^ for *Arabidopsis* [5], 6.5 × 10^−9^ for *B. distachyon* [44], 1.4 × 10^−8^ for *B. rapa* [45], 6.1 × 10^−9^ for *G. max* [44], 6.5 × 10^−9^ for *O. sativa* [46], 9.1 × 10^−7^ for *P. trichocarpa* [47], 1.5 × 10^−8^ for *G. raimondii* [48], and 6.5 × 10^−9^ for *Z. mays* [49]. The Phytozome database (http://www.phytozome.org) was used to identify tandem duplication gene pairs. On the same chromosome, if the TF family members are contained within ten genes before and after a homologous gene, it is proved that these two homologous genes resulted from tandem duplication [41].

### 2.4 Functional divergence, positive selection, and coevolution analyses

The DIVERGE software (version 3.0) was used to detect the Type I and Type II functional divergence sites in different groups of the *GRF* gene family through posterior analysis [50]. The Type I and Type II functional divergence coefficients (*θ*_I_ and *θ*_II_) between members of a subfamily were obtained to measure the degree of divergence, such that if *θ*_I_ or *θ*_II_ were significantly greater than 0, then it can be said that certain amino acid sites underwent significant changes in their evolution rates or physiochemical properties, respectively [51,52]. The *Q*_*k*_ value is an important indicator for measuring the degree of functional divergence at the amino acid site and is directly proportional to the probability of any functional divergence between two subfamilies [52]. In this study, the critical value of *Q*_*k*_ was set to 0.8.

Positive selection was investigated using a maximum-likelihood approach using site models and branch site models in the CODEML program of PAML v4.4 [53,54]. Comparing the two models, null models (M0 and M7) and alternative hypothesis models (M3 and M8) were executed for positive selection identification. A likelihood ratio test (LRT) was performed according to the chi-square distribution, and then the alternative hypothesis model was established based on the *p* value. The Bayes empirical Bayes (BEB) method was used to calculate the posterior probabilities [55].

The coevolution amino acid site was calculated by coevolution analysis using protein sequences with the PERL software. BLOSUM-corrected amino acid distances were used to identify amino acid covariations [56].

### 2.5 Protein structure prediction

Construction of the 3D structure of GRF proteins was done using the online website PHYRE2 (http://www.sbg.bio.ic.ac.uk/phyre2/html/page.cgi?id=index) [57,58], and important amino acid sites on the 3D structure were marked using the PyMOL v1.7.4 software (Schrödinger, Inc.).

### 2.6 Expression analysis of GRFs

RNA-seq data of four different plant species were obtained from the following websites: *Arabidopsis* eFP Browser (http://bar.utoronto.ca/efp/cgi-bin/efpWeb.cgi) [59], soybean eFP Browser (http://bar.utoronto.ca/efpsoybean/cgi-bin/efpWeb.cgi) [60], rice eFP Browser (http://www.bar.utoronto.ca/efprice/cgi-bin/efpWeb.cgi) [61], and *Brachypodium* eFP Browser (http://bar.utoronto.ca/efp_brachypodium/cgi-bin/efpWeb.cgi). Then, the GenePattern (http://www.broadinstitute.org/cancer/software/genepattern/) software tool was used to construct the expression heat map.

## 3 Results

### 3.1 Genome-wide identification of *GRF* gene family members

Sequences of nine *GRF* gene family members in the *Arabidopsis* genome were obtained from the TAIR database and used as seed sequences. Using the BLASTP tool in the Phytozome database, 173 members of the *GRF* gene family were identified in 15 species. In addition, results show that the values of pIs ranged from 4.78 to 10.57 (Table S1), with an average value of 8.12, and that 79.7% of GRF protein members were weakly alkaline, whereas only 20.3% were weakly acidic. The number of amino acid residues was between 155 and 817, with an average of 406, and the MW was between 22.555 and 86.039 kDa. These data show that to adapt to changes in the external environment and meet the functional requirements of different periods in the long-term evolution process, members of the *GRF* gene family underwent changes in their respective physical and chemical properties.

### 3.2 Phylogenetic relationships and molecular characterization of *GRF* gene family

**Figure 1:**
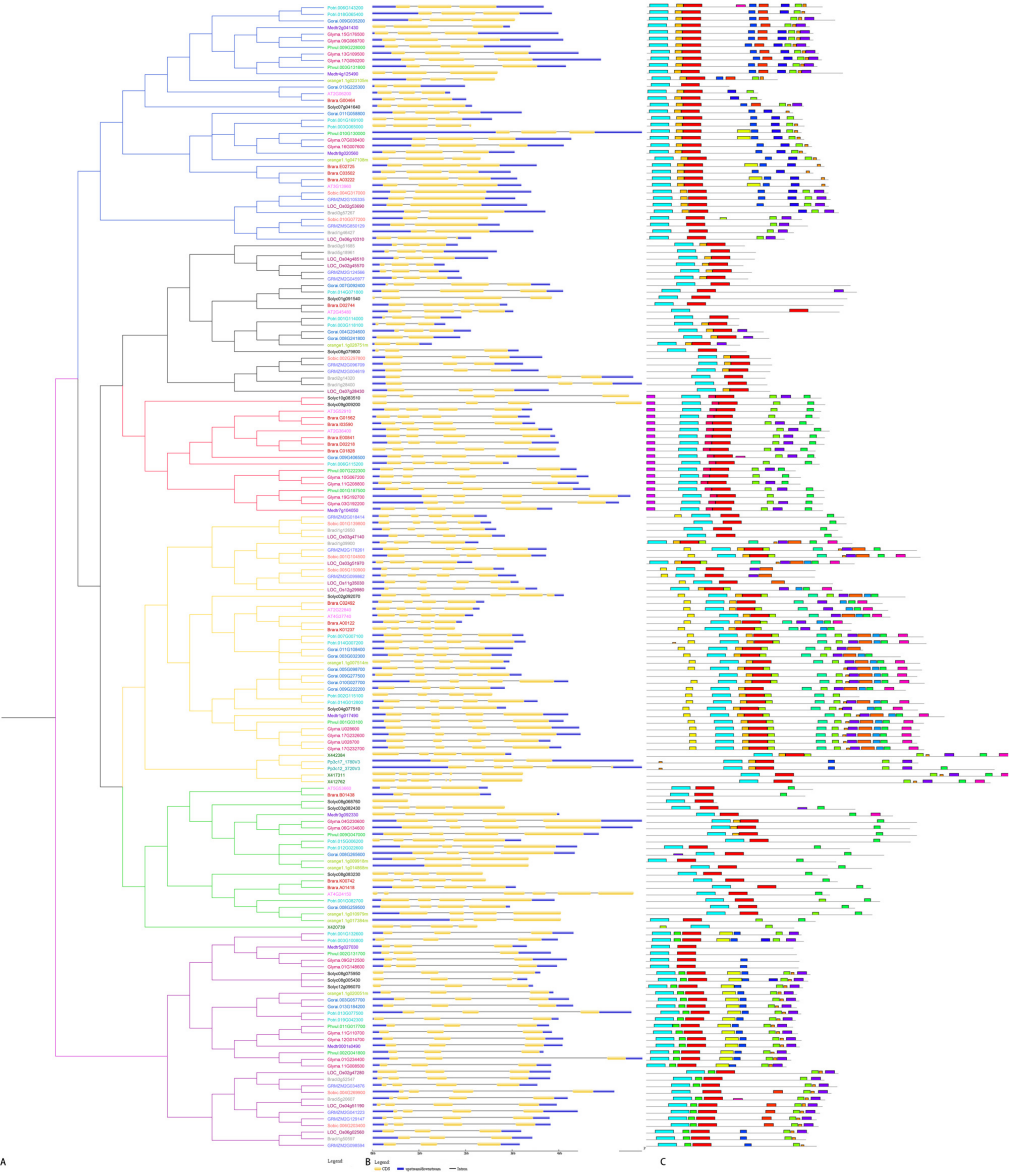
Phylogenetic relationships and exon–intron structures of *GRF* gene family members: (a) Bayesian phylogenetic tree of the *GRF* gene family. The color of subclades indicates the six corresponding gene subfamilies. Blue, black, red, yellow, green, and purple represent Groups I, II, III, IV, V, and VI, respectively. Gene names of different species are represented by different colors. (b) Exon–intron structures of the *GRF* genes. Yellow bars: exons; lines: introns; blue bars: 3′ untranslated region. (c) Distributions of conserved motifs. Twenty putative motifs are indicated in different colored boxes.

**Figure A1:**
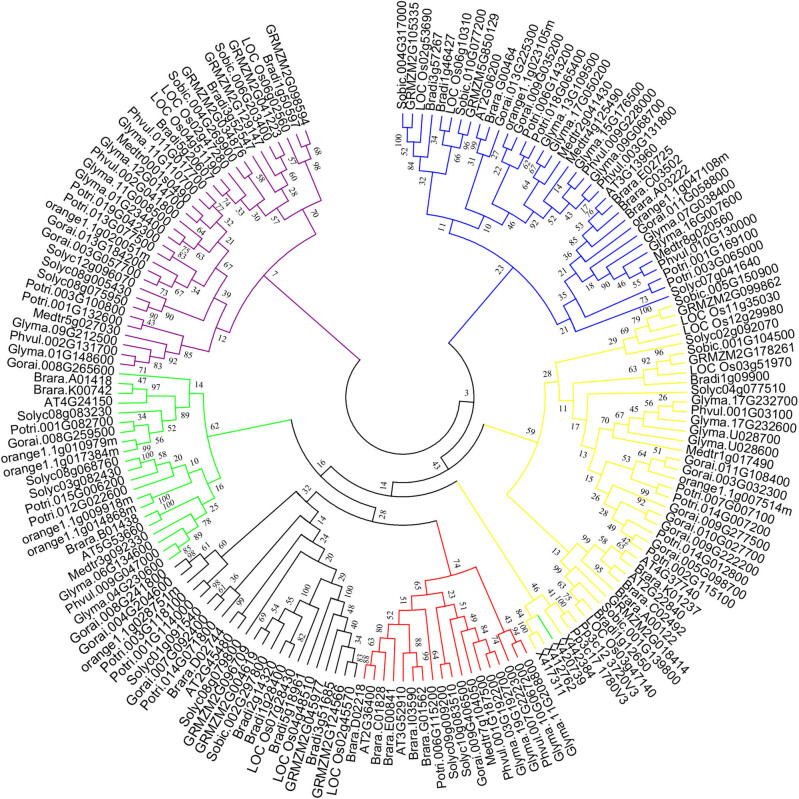
NJ phylogenetic tree of all the *GRF* gene family. Blue, black, red, yellow, green, and purple represent Groups I, II, III, IV, V, and VI, respectively.

**Figure A2:**
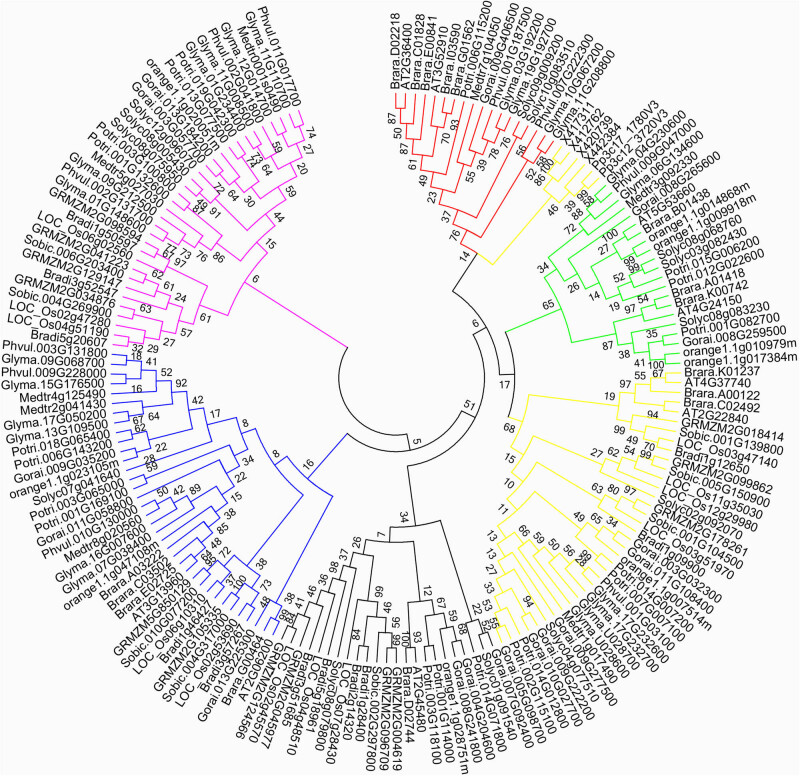
ML phylogenetic tree of all the *GRF* gene family. Blue, black, red, yellow, green, and purple represent Groups I, II, III, IV, V, and VI, respectively.

Multiple sequence alignment of the 173 proteins identified was performed, and a NJ phylogenetic tree (Figure A1), maximum-likelihood phylogenetic tree (Figure A2), and a Bayesian phylogenetic tree (Figure 1a) were constructed. Comparative analysis showed that the three trees had similar topological structures, proving the reliability of phylogenetic tree results, and the Bayesian tree was used for subsequent analysis. Phylogenetic analysis divided the *GRFs* into six subfamilies, namely Groups I, II, III, IV, V, and VI, containing 36, 23, 18, 41, 22, and 33 members, respectively. Moreover, phylogenetic tree analysis showed that Groups I, II, IV, and VI contained both monocots and eudicots (Figure 1a), whereas Group III only contains eudicots. Except for one member of Group V is *Selaginella*, all the others are eudicots. Pteridophyta and bryophyta are mainly distributed in Group IV. Based on the structural characteristics of the phylogenetic tree and the analysis of subfamily members, it can be deduced that all family members have a common ancestor. During the evolution process, the GRF family members, including lower plants, differentiated first and evolved into the Group IV. The differentiation of Group III and V was thought to have occurred after the monocots and eudicots split. The evolutionary pattern of GRF TFs was significantly different between monocots and eudicots.

**Figure 2:**
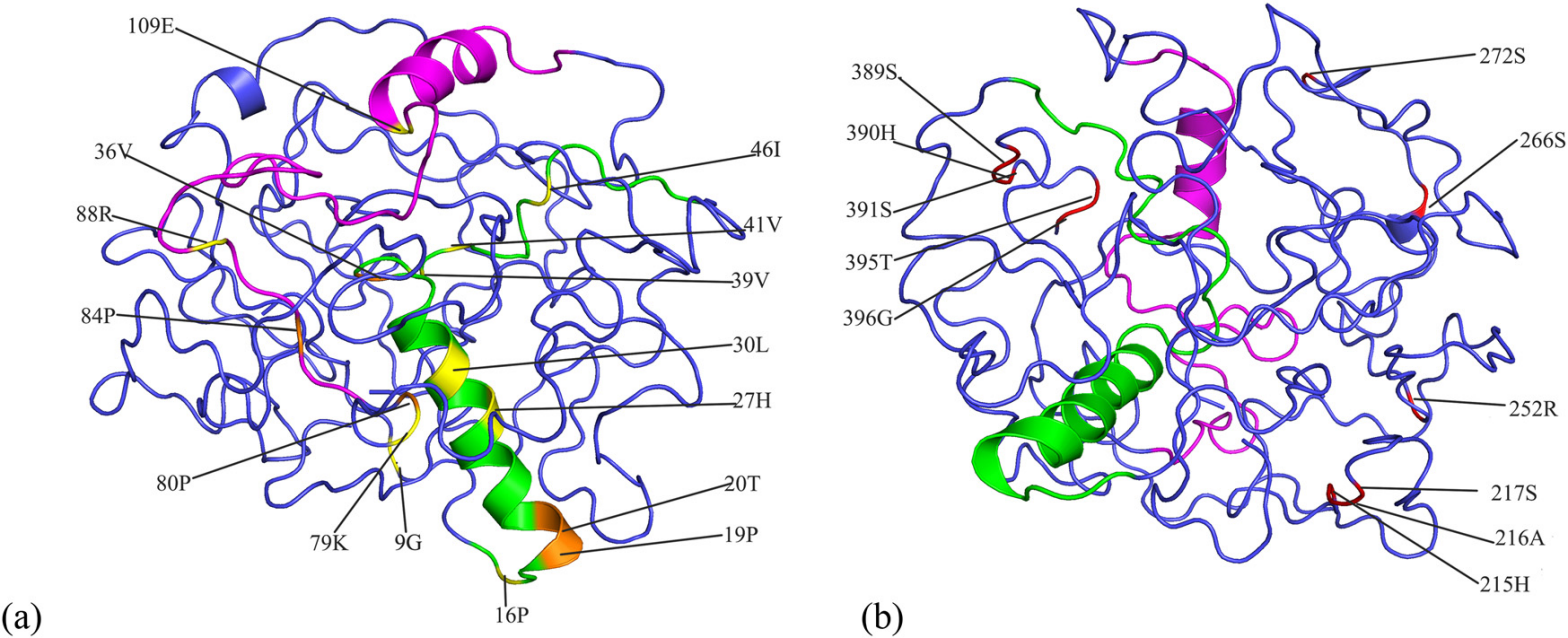
Schematic of 3D structure of plant GRF protein: (a) 3D structure of AT3G13960. The QLQ and WRC domains are colored in green and magenta, respectively. Yellow indicates Type I functional divergence sites and orange indicates positive selection sites. (b) The sites responsible for the coevolution sites are colored in red.

**Figure 3:**
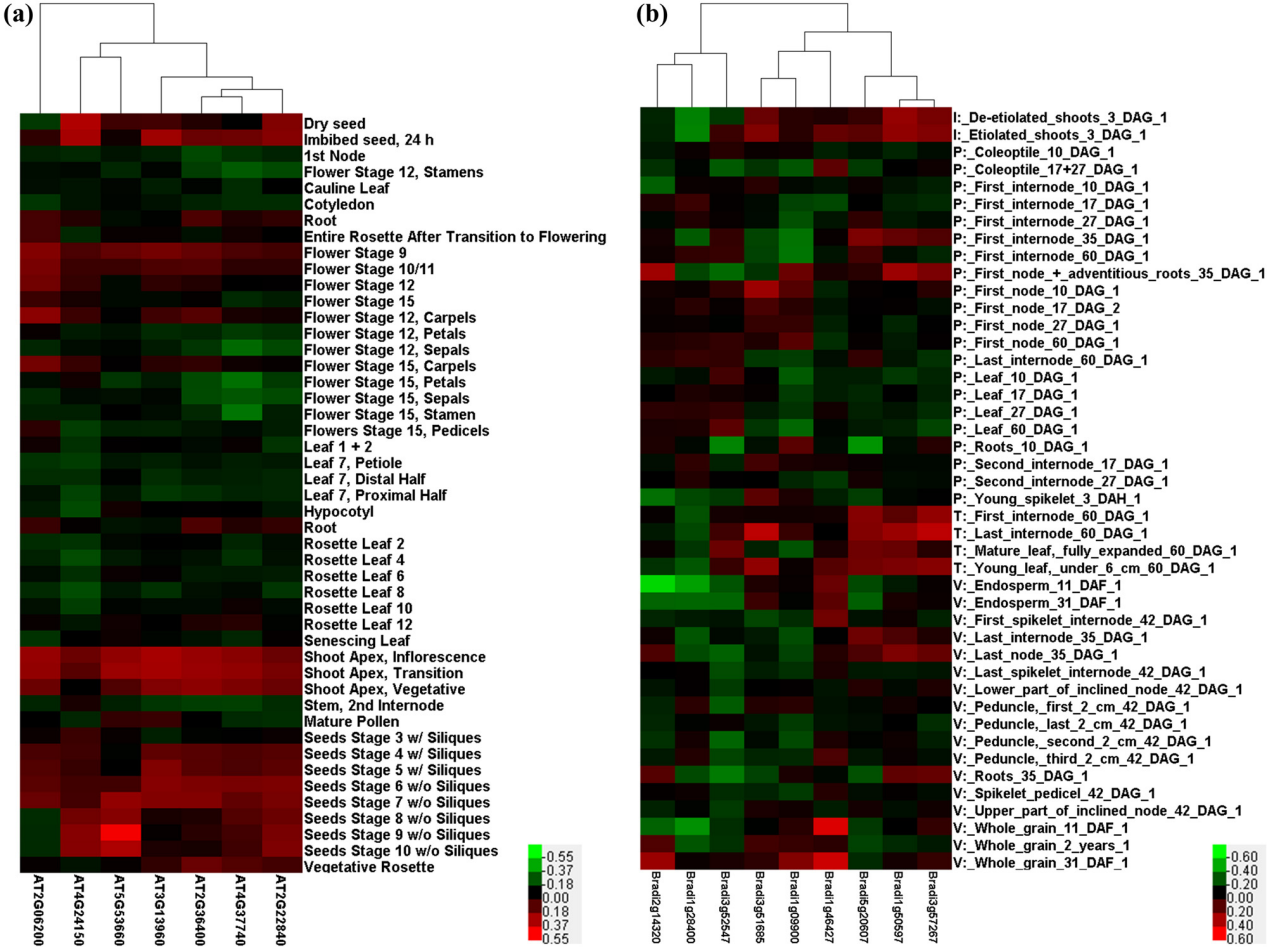
Expression profiles of (a) *A. thaliana* and (b) *B. distachyon GRF* genes. Reddest (hot) and greenest (cool) shades denote higher and lower expression levels, respectively. Gray indicates the value of 0 in the original RNA sequence data, and the software automatically recognizes this part of the data as “missing.”

The results of exon–intron analysis (Figure 1b) showed that most members of Groups I, II, and VI were composed of three exons, and other members contained two or four exons. In Groups III, IV, and V, most members contained four exons, and a few members, such as *X417311*, *X412762*, and *AT4G24150*, contained six exons. Another member, *Solyc08g068760*, contained only one exon. The results of exon–intron further prove the reliability of the phylogenetic tree branch. Differences in the number of exons may have been due to the loss or acquisition of exons during long-term evolution. Furthermore, the number of exons in higher plants decreased, which may be due to the loss of introns.

The potential motif structures in the GRF family were obtained, and 20 conserved motifs, designated as motifs 1–20, were identified (Figure 1c). Members of the same subfamily contained a similar number and sequence of motif types. All members had motifs 1 and 2, which contained the WRC and QLQ domains, respectively. In addition, the highest numbers and types of motifs were observed in Group IV, including the bryophyta, pteridophyte, monocots, and eudicots. It is worth noting that different subfamilies contained unique motifs; Group I contained motifs 7, 10, and 14; Group III contained motif 15; moreover, motifs 19 and 20 were present in Group IV, and motif 11 was specific to Group VI. This may be related to the functional divergence among various subfamilies during evolution, supporting the classification.

**Figure 4:**
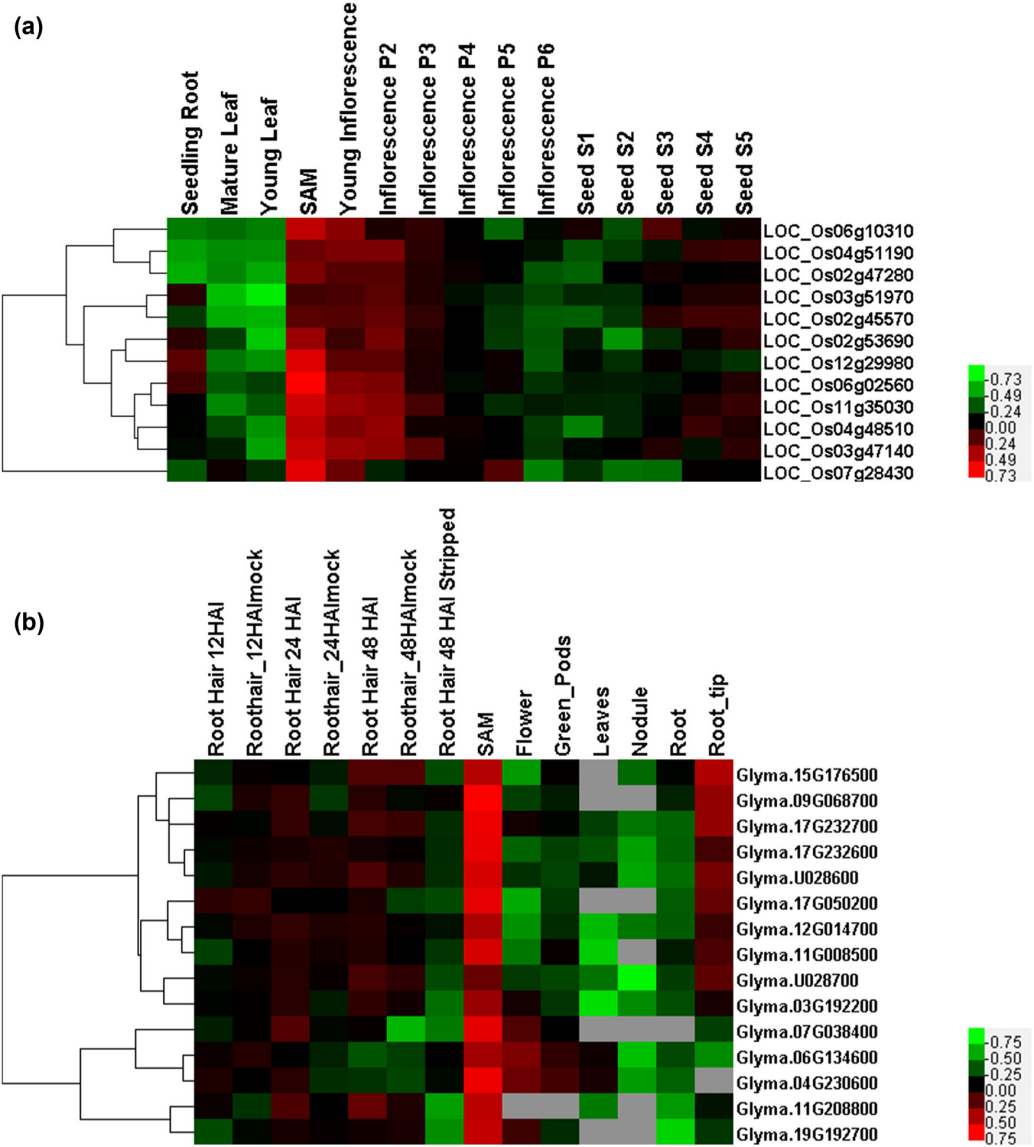
Expression profiles of (a) *O. sativa* and (b) *G. max GRF* genes. Reddest (hot) and greenest (cool) shades denote higher and lower expression levels, respectively. Gray indicates the value of 0 in the original RNA sequence data, and the software automatically recognizes this part of the data as “missing.”

### 3.3 Analysis of *cis*-acting elements in the promoter region of *GRF* genes

To better understand the expression and function of the GRFs, seven types of *cis*-acting elements in the promoter region of the *GRF* gene family were identified, namely light-responsive elements, development-related elements, hormone-responsive elements, environmental stress-related elements, site-binding related elements, promoter-related elements, and other elements. Among them, light-responsive elements, development-related elements, hormone-responsive elements, and environmental stress-related elements were closely related to plant growth regulation.

We analyzed light-responsive *cis*-acting elements, such as Box4, GT1-motif, G-box, ATCT-motif, and TCT-motif, which was the most abundant type in the *GRF* gene family (Table S2). Box4, G-box, GT1-motif, and TCT-motif were abundant, with average copies of 1.74, 1.12, 0.91, and 0.66, respectively. For example, G-box has widely existed in the promoters of light-controlled genes and other environmental factors regulating genes. It has a highly conserved core sequence CACGTG, a universal regulatory element for plants responding to external environmental stimuli. Hormone responsive elements, including the GARE-motif, TCA-element, CGTCA-motif, TGACG-motif, and abscisic acid-responsive element (ABRE), which are involved in response to gibberellin, auxin, methyl jasmonic acid, and abscisic acid, have also been identified. Of these, the most abundant was ABRE, and the average copies of ABRE, ERE, CTCCA-motif, and TGACG-motif were 1.52, 1.29, 0.95, and 0.94, respectively. In addition, CCGTCC-box (12.37%), CAT-box (14.68%), and O_2_-site (12.58%) were identified as the development-related elements; CCGTCC-box participated in the development of meristems. Meanwhile, we identified the *cis*-acting elements in response to environmental stress: LTR, MBS, WUN-motif, GC-motif, ARE, and TC-rich repeats. The WUN-motif, TC-rich repeats, and LTR were damage response elements, stress response elements, and low-temperature response elements, respectively, indicating that the *GRF* genes were related to defense recovery and temperature change response. The existence of these elements indicates that a variety of environmental factors may regulate *GRF* gene expression.

In addition, the composition of *cis*-acting elements between different subfamilies is not only similar, but also differentiated. Several types of *cis*-acting elements have a large distribution among each subfamily, such as G-box, ARE, as-1, ERE, and ABRE, and they have different functions. ARE is an essential *cis*-acting regulatory element involved in anaerobic induction. The difference in *cis*-acting elements in different subfamilies also supported the differentiation of *GRF* genes between different subfamilies, thus promoting functional divergence during evolution.

### 3.4 Gene duplication event of GRFs

Gene duplication is one of the main mechanisms for the establishment of new gene functions and biological evolution. It is well known that gene duplication can occur in multiple ways, including segmental duplication, tandem duplication, and transposition events, which provide raw materials for evolutionary mechanisms [62]. In this study, we mainly focused on segmental and tandem duplication. Tandem duplication events usually produce multiple family members within the same or adjacent intergenic regions [63]. We only found two pairs of tandem duplication genes from soybeans (*Glyma.17G232700* and *Glyma.17G232600*; and *Glyma.U028700* and *Glyma.U028600*), which were members of Group IV. This showed that tandem duplication accounted for a small proportion of the evolution of the *GRF* gene.

In addition, 40.5% of the *GRF* gene family members were associated with segmental duplication events (Table S3). Segmental duplication events were most active in eudicots. Among *Arabidopsis*, cabbage, soybean, cotton, and poplar, a total of 51 genes were confirmed to be segment duplicated genes. Segmented duplication events of eudicots were distributed in all groups, whereas segmented duplication events in monocotyledonous plants were mainly concentrated in Groups, I II, and VI. Considering together, our results suggest that segmental duplication promoted the expansion of the *GRF* gene family. Interestingly, segmental and tandem duplication events were both found in Group IV, suggesting that both types of duplications contributed to the expansion of Group IV. In parallel, both genes in each duplicate gene pair belonged to the same group. These genes might be not undergoing functional divergence during the evolution process.

Large-scale duplication events generate a large number of homologous genes. Table S3 lists the average *K*_s_ values (synonymous base substitution rates) and estimated dates for the segmental duplication events of the *GRF* gene family in eight plant species. As shown in the table, most of the segment duplication gene pairs should have been generated and retained along with whole-genome duplications (WGDs), such as *Arabidopsis*, *G. raimondii*, *O. sativa*, and *B. rapa*. In some species, such as *P. trichocarpa* and *Z. mays*, partial segment duplication events are earlier than WGDs event. It is speculated that the remaining segment duplication genes may have originated from independent repeat events. In the process of gene replication, mutations may occur, leading to functional divergence and species diversification among the *GRF* gene subfamily members.

### 3.5 Functional divergence in the *GRF* gene family

To further analyze whether amino acid substitutions in the *GRF* gene family lead to functional divergence, the Type I functional divergence coefficient (*θ*_I_) between two groups was detected by posterior analysis using DIVERGEv3.0 [50]. Results ranged from 0.023 to 0.689 and were significantly greater than 0 (Table 1). Except for Groups II and III, and II and IV, the LRT values among other groups reached extremely significant levels (*p* < 0.01), which indicated that plant GRF protein had sites that underwent Type I functional divergence during the evolutionary process. A similar analysis showed no Type II functional divergence sites related to the physicochemical properties of amino acid residues. The degree of type II functional divergence (*θ*_II_) is not significantly greater than 0, and the detected sites are not statistically significant.

**Table 1:** Functional divergence sites among groups of the *GRF* gene family

Group 1	Group 2	Type I			Type II	
		*θ*_I_ ± s.e.	LRT	*Q*_*k*_ > 0.8	*θ*_II_ ± s.e.	*Q*_*k*_ > 0.8
I	II	0.265 ± 0.071	5.394*	41V	−0.155 ± 0.202	None
I	III	0.337 ± 0.125	4.200*	39V	−0.058 ± 0.153	None
I	IV	0.188 ± 0.075	1.246*	None	−0.096 ± 0.153	None
I	V	0.427 ± 0.082	16.799**	27H,30L,46I,9K,88R	−0.020 ± 0.183	None
I	VI	0.181 ± 0.183	0.408	None	−0.045 ± 0.130	None
II	III	0.023 ± 0.022	0	None	−0.262 ± 0.228	None
II	IV	0.289 ± 0.098	6.100*	41V	−0.120 ± 0.210	None
II	V	0.159 ± 0.107	0.013	None	−0.163 ± 0.254	None
II	VI	0.030 ± 0.022	0	None	−0.247 ± 0.204	None
III	IV	0.036 ± 0.113	7.255*	39V,109E	−0.031 ± 0.158	27H,113H
III	V	0.369 ± 0.088	4.291*	39V,109E	−0.038 ± 0.188	27H
III	VI	0.182 ± 0.220	2.418*	None	0.009 ± 0.135	None
IV	V	0.057 ± 0.100	1.244*	None	−0.038 ± 0.194	None
IV	VI	0.635 ± 0.192	6.270*	16P,39V	−0.047 ± 0.134	113H
V	VI	0.689 ± 0.171	7.919*	27H,46I,79K	0.022 ± 0.171	None

Note: *θ*_I_ and *θ*_II_: the coefficients of Type-I and Type-II functional divergence. LRT: likelihood ratio test. *: *p* < 0.05, **: *p* < 0.01, highly significant. *Q*_*k*_: posterior probability.

By calculating the posterior probability of each site (*Q*_*k*_ > 0.8), the key amino acid sites related to functional divergence of the *GRF* gene were determined. The results revealed 10 Type I functional divergence sites (9G, 16P, 27H, 30L, 39V, 41V, 46I, 79K, 88R, and 109E). Of these sites, simultaneous changes in the evolution rate and physicochemical properties of site 27H were observed. Our results suggest that the functional difference between any two groups was mainly due to the difference in their amino acid evolutionary rates.

### 3.6 Positive selection, coevolution, and three-dimensional structural analysis of the *GRF* gene family

Site-specific models for positive selection were used to determine sites that underwent positive selection during evolution. By comparing M0 (one ratio) and M3 (discrete) models, we calculated the twice log-likelihood difference of the models and obtained 2ΔlnL = 932.8146 (*p* < 0.01, d*f* = 4). This LRT result was statistically significant, indicating that the amino acids in the *GRF* gene family have experienced variable selection pressures among sites. Comparing the M7 (beta) and M8 models (beta and *ω*), the 2ΔlnL was 5795.66, and the *ω* value implanted in the M8 model was 2.55821, which was much greater than 1 (Table 2). Then, the BEB was used to evaluate the posterior probability of the location considered to be a positive choice. A total of six positively selective sites were detected in the M8 model, and their posterior probabilities were all greater than 0.95. Of these sites, one (36V; *p* < 0.05) was at a significant level, and five (19P, 20T, 39V, 80P, 84P; *p* < 0.01) were at an extremely significant level (Table 2).

**Table 2:** Positive selection test of plant GRFs using site-specific models

Model	In*L* ^a^	2Δln*L*	Estimate of parameters	Positively selected sites^b^
M0	−12532.1472	932.8146**	*ω* = 0.08401	Not allowed
M3	−12065.7399		*p*0 = 0.36332, *p*1 = 0.42999, *p*2 = 0.20668, *ω*0 = 0.00414, *ω*1 = 0.08165, *ω*2 = 0.28340	None
M7	−12032.7693	5299.5134**	*p* = 0.41819, *q* = 3.82194	Not allowed
M8	−14682.5260		*p*0 = 0.99999, *p* = 0.71594, *q* = 1.59678, *p*1 = 0.00001, *ω* = 2.55821	19P**, 20T**, 36V*, 39V**, 80P**, 84P**

Note: a: log likelihood. b: Positive selection sites are inferred at posterior probabilities >95%. *: *p* < 0.05, **: *p* < 0.01.

We identified four groups of coevolution sites in the *GRF* gene family: 215H and 216A; 217S and 272S; 395T and 396G; and 252R, 266S, 391S, 390H, and 389S. These sites were distributed at the C-terminus with transcriptional activity and were not far apart in the tertiary structure, indicating that their compensatory mutations contributed to local stability maintenance.

The *GRF* gene family member *AT3G13960* from *Arabidopsis* was used as a representative to construct the 3D structure. As shown in Figure 2a, the identified positive selection sites marked on the 3D structure were mostly distributed on the QLQ and WRC domains, and three sites were placed on the alpha helix. These results indicate that the positive selection sites have undergone adaptive evolution in the process of evolution and may also play an important role in maintaining the stability of the protein structure. Similarly, the Type I functional divergence sites were also mainly distributed in the QLQ and WRC domains (Figure 2a). We speculate that these sites may be involved in protein–protein interactions. The detected coevolution sites were mainly distributed at the C-terminus of the GRF protein (Figure 2b), and they are related to each other during the evolution process as one of the sites cannot be mutated alone. The C-terminus of GRF protein has transcriptional activity, suggesting that these amino acid sites may play a vital role in maintaining protein transcriptional activity, which reflects the conservation of protein structure and function.

### 3.7 Expression profiling of *GRF* genes

To analyze the function of the *GRF* gene and explore its expression in different organs and developmental stages, we selected four species (*A. thaliana*, *O. sativa*, *G. max*, and *B. distachyon*) for expression profile analysis using published RNA-seq data. All members of the *GRF* gene family in rice (Figure 4a) have the characteristic upregulated expression in the stem apex meristem (SAM), as well as in soybean *GRF* gene family members (Figure 4b), indicating that *GRF* gene family members have conservative characteristics and functions related to growth and development in SAM. In addition, different subfamily members from the same species have different expression profiles in different developmental tissues. For example, the *GRF* gene family members from *Arabidopsis* (Figure 3a) have different expression profiles in flowers, leaves, shoots, and seeds, and with especially high expression in shoots and seeds. Moreover, the expression profiles at different developmental stages of the same tissue were slightly different, and expression level in the early stages was higher than that in the later stages. The *GRF* gene family members *LOC_Os11g35030* and *Glyma.17G232600* from Group IV both exhibited high expression characteristics in SAM, and both contained *cis*-regulatory elements called as-1 that are involved in root-specific expression, and are closely related to root growth and development. Analysis of the expression profiles of the four plants showed that the expression of *GRF* gene members in different subfamilies was similar and differentiated among members.

## 4 Discussion

### 4.1 Molecular characterization and genomic analysis of the *GRF* gene family

Genome-wide analysis of 15 species identified 173 TF family members, and the *GRF* gene family was divided into six groups, namely Groups I–VI, by phylogenetic analysis (Figure 1a). The distribution of each species was different; only *P. trichocarpa*, *G. raimondii*, and *S. lycopersicum* were common in the six groups. This is different from the five subfamily classifications of Cao et al. [64], probably, because there are more species used in our research included lower and higher plants represented all of the plant kingdom. However, the number of introns and exons in each group was similar, suggesting that *GRF* structure was conserved within a branch. The *GRF* gene-coding region of eudicots was mainly composed of four exons, whereas that of monocotyledonous plants was mainly composed of three exons (Figure 1b). Moreover, results showed that 20 motifs were detected, with at least one or two conserved motif types and spatial arrangement patterns in the same subfamily. All GRFs contained motifs 1 and 2 (Figure A2), which meant that the WRC and QLQ conserved domains at the N-terminal region were present in all screened GRF proteins. The GRF protein interacts with the GIF protein through the QLQ domain, forming a functional complex with a transcriptional activation function. Earlier studies have shown that in *Arabidopsis*, compared with other AtGRF family members, *AtGRF5* (*AT3G13960*) binds to AtGIF more tightly and plays a greater role in cell proliferation in the leaf primordia [65]. In brief, there are obvious differences between each group, and the functions of GRF members within the same subfamily have certain similarities. These special domains may specifically combine with other molecules to function.

Gene duplication events or the formation of new species can lead to diversification of protein functions. The functional difference between duplication genes is caused by the accumulation of repeated mutations at amino acid sites [66]. According to our results, 45 pairs of segmental duplication genes were detected in eight species (Table S3), and only two pairs of tandem duplication genes were found in soybean, indicating that the amplification of the *GRF* gene family mainly relied on segmental duplication, and these segmental duplication genes can be retained in different species through whole genome duplication events, which was consistent with the study of Chen et al. in soybeans [67].

In addition, according to the research of Fonini et al., it is speculated that the earliest duplication event of *GRF* gene was the replication event in the common ancestor of charophyte and land plants [68].

By analyzing the functional divergence of the *GRF* gene family, ten Type I functional divergence sites were detected in this experiment, indicating that changes in the evolution rate of amino acid sites was the main driving force for the functional differentiation of the *GRF* gene family. Phylogenetic analysis has confirmed that positive selection contributes to protein evolution, and that changes in positive selection sites allow proteins to acquire new catalytic functions without injuring their main biochemical properties [69]. Six sites of positive selective (19P, 20T, 36V, 39V, 80P, 84P; Table 2) in the *GRF* gene family were revealed, and most of these sites were located in the conserved domains (Figure 2a), suggesting that they may play an indispensable role in the interaction with the (SNH) domains of the plant GIF protein. We speculate whether the changes in these positive selection sites may interfere with the formation of complexes between GRF and GIF proteins, thus affecting the activation of GRF protein, and the growth and shape of leaves and petals [15].

It was found that there was an amino acid site (39V) that experienced both Type I functional divergence and positive selection. The change in the evolutionary rate of this amino acid site was also favorable for alleles to improve fitness, and this may be one of the important evolutionary forces for functional divergence after gene duplication. It is believed that this amino acid site may have a substantial role in maintaining the stability of the GRF protein.

The presence of intramolecular coevolutionary networks is also one of the factors that determine the evolution of proteins. The complexity of evolution is related to the potential functional and structural interactions between sites [70]. Four groups of coevolution sites were found to be located at the C-terminus of the GRF protein (Figure 2b). The C-terminal domain is more functionally diverse compared to the conserved N-terminal domain [11]. Wu et al. found that although *ZmGRF10* contains a complete N-terminal domain, its transcriptional activation activity is lost due to the lack of a complete C-terminal domain, thus affecting plant growth [6]. We suspect that these coevolution sites at the C-terminal domain will affect the transcriptional activity of the GRF protein, which is worthy of further investigation. These coevolution sites reflect the conservation of protein functions or structures.

### 4.2 Expression and potential functions of GRFs

According to the analysis of phylogenetic trees obtained from multiple species, *GRF* family members with high homology and similar structures usually clustered together. Therefore, the function of known *GRF* gene family members can be used to predict the function of unknown members in the same branch. Tissue-specific expression profiles may also be similar among different species in the same subfamily. Among the members of GRFs expressed in flower organs and meristems, *AtGRF7*, *8*, and *9* (*AT5G53660*, *AT4G24150*, *AT2G45480*) are involved in pistil development, and *AtGRF8* is particularly important in the late development of floral organs [13,21,24]. According to the expression profiles obtained, it can be hypothesized that the soybean family members *Glyma06G134600* and *Glyma04G230600* that belonged to Group V with *ATGRF7* and *8*, were also highly expressed in flowers (Figures 3a and 4b). We believe that they also played a role in the development of soybean flower organs. Studies have shown that the expression of *AtGRF7* is related to the regulatory mechanism of abscisic acid-responsive elements (ABRE). AREBs/ABFs can activate DREB2A transcription through ABRE in response to osmotic stress, whereas *AtGRF7* can bind to the promoter region of DREB2A to inhibit osmotic stress or abscisic acid response and prevent growth inhibition [13]. Soybean GRFs that are part of Group V also contain ABRE *cis*-acting elements, which are speculated to be related to osmotic stress and abscisic acid response [71]. In addition, the research on abiotic stress of soybean found that the morphology of soybean under shading conditions has undergone great changes, including reduced leaf area and weight, and excessive elongation of stems [67]. As mentioned earlier, many light-responsive regulatory elements have been detected in the GRF gene. We speculate that shaded conditions affect the expression of light-responsive elements in *GRF* gene and have an impact on the growth and development of soybeans.

Based on the expression profiles, it was found that most *GRF* members in rice are usually highly expressed in SAM and young inflorescences. *OsGRF1*, which was preferentially expressed in the stem apex containing the SAM and the younger leaf primordia, can regulate the growth of leaves. Another member of Group I, *LOC_Os06g10310*, showed a trend of upregulation in SAM (Figure 4a). Similarly, in soybeans, different *GRF* subfamily members were highly expressed in the meristems, and analysis of *cis*-acting elements showed that most members contained *cis*-acting regulatory elements related to meristem expression and meristem specific activation, such as CAT-box and CCGTCC-box1. At the same time, according to the research on other plants, the expression of *GRF* gene in wheat shoot tip meristem is significantly higher than that in other tissues [72], In tobacco, it is found that *NtabGRF* gene is highly expressed in active growing tissues and responds to various hormone treatments [73]. All results confirmed that the *GRF* gene is highly expressed in the vigorously divided tissues of plants, and it is speculated that the *GRF* gene plays an important role in the early stage of plant growth and development. We hypothesize that the expression similarity of genes in different tissues of the same species is greater than their expression similarity in the same tissue of different species.

However, it can be seen that *Arabidopsis GRFs* in different subfamilies also have different expression profiles in the same tissues. *AtGRF3* and *1*, and *AtGRF2* from Groups III and IV, respectively, were highly expressed in the roots, whereas *AtGRF7* and *8* from Group V were relatively downregulated in roots. This may have been caused by functional divergence during evolution. In addition, *GRF* regulates root growth through MIR396a, which affects the extension zone and regulates root growth. It was also found that *B. distachyon* GRFs from Groups I and VI (*Bradi3g57267*, *Bradi1g46427*, *Bradi5g20670*, *Bradi1g50597*, and *Bradi3g52547*) were highly expressed in young leaves, whereas the members of Groups I and VI in soybean showed a trend of decreased expression in young leaves; they also did not have similar expression profiles in similar tissues (Figures 3b and 4b). This may also indicate functional divergence caused by gene duplication during the evolutionary process.

## 5 Conclusion

Genome-wide analysis of 15 plant species identified 173 members of the *GRF* gene family, which were divided into six subfamilies. The molecular structure characteristics, phylogeny, gene duplication, and expression patterns in different tissue analyses revealed an evolutionarily conserved transcriptional activity of the *GRF* gene family. Type-I functional divergence was identified as the main reason for the functional diversification of GRFs, and positive selection sites played an important role in domain differentiation. The appearance of multiple *cis*-acting elements indicated that GRFs were regulated by diverse hormones and environmental factors. Members of the same subfamily contained similar *cis*-acting elements, and their expression profiles reflected the conservation of *GRF* gene family members; however, the differences of *GRFs* between species also reflected the differentiation of *GRF* gene family members during evolution. This study provides useful information for further exploration of the molecular evolution mechanism and functional features of the plant *GRF* gene family.
